# Supplementary material for: Signal-strapping as a protein-sequence search method for the discovery of metalloproteins
Source: Nat Commun. 2025 Oct 20;16:9244. doi: 10.1038/s41467-025-64309-x (PMC12537917; doi:10.1038/s41467-025-64309-x)
Supplement: Supplementary file 1 — Supplementary Information [file 41467_2025_64309_MOESM1_ESM.pdf]

SUPPLEMENTARY INFORMATION

Signal-strapping as a protein-sequence search method for the discovery of metalloproteins

João Paulo L. Franco Cairo,[1] Thamy L. R. Corrêa,[1] Wendy A. Offen,[1] Alison K. Nairn,[1] Julia Walton,[1] Sean T. Sweeney,[2]* Gideon J. Davies[1]* and Paul H. Walton[1]*

1. Department of Chemistry, University of York, Heslington, York, UK, YO10 5DD.
2. Department of Biology, University of York, Heslington, York, UK, YO10 5DD

*List of corresponding authors
Sean T. Sweeney: sean.sweeney@york.ac.uk
Gideon J. Davies: gideon.davies@york.ac.uk
Paul H. Walton: paul.walton@york.ac.uk

Supplementary Discussion

Oxidoreductase activities of identified metalloproteins.
To investigate the functional potential of the newly reported enzymes, a series of enzymatic assays were performed to determine whether their metal sites could catalyse redox chemistry, as expected of transition-metal-based enzymes. Copper-, Nickel-, and Cobalt- loaded forms of *Is*DUF4198, *Pa*DUF6702, *Rb*Ang-1a, and *Mm*Ang-2a were evaluated for oxidase and peroxidase activities using the Amplex®Red/HRP[1] and 2,6-Dimethoxyphenol (DMP)[2] methods respectively. Copper loaded forms of *Is*DUF4198, *Pa*DUF6702, *Rb*Ang-1a, and *Mm*Ang-2a were able to perform oxidase activities at yield nearly to that observed for free $CuCl_2$ as expected,[3] while the well-known LPMO *Ls*AA9a from *Lentinus similis*[4] had very low oxidase activity. Ni-loaded *Is*DUF4198 performed very weak oxidase activity, however, at the same level as for free $NiCl_2$. No significant oxidase activity was found to the cobalt-loaded form of the target proteins.

Peroxidase activity assays revealed that the copper loaded forms of the all targeted proteins performed very low or no peroxidase activities when compared to known LPMOs such as *Ls*AA9a and AA9 LPMO AN6428[5] from *Aspergillus nidulans*. However, those low peroxidase activities is also consistent with some LPMOs, particularly from AA10 families.[2] Ni-*Is*DUF4198 also presented very low peroxidase activity and none of the cobalt-loaded forms of the targeted proteins performed peroxidase activity. Cu-*Pa*DUF6702 was also tested for protease activity but showed no activity with substrates like Azocoll, Keratin azure, or Azocasein, even with ascorbate or extended reaction times, suggesting a non-specific metal binding and transfer function. None of the copper loaded metalloproteins described herein catalysed the oxidative cleavage of polysaccharides.

Detail statistic of the metal binding site of the AlphaFold 3 model of *Pa*DUF6702.
The ipTM score was calculated for both the Cu- and Co-loaded forms of *Pa*DUF6702, showing high scores (> 0.8) (Supplementary Fig. 4 and 5). Based on these predictions, the bond lengths between the metal and the four nearby nitrogen atoms (NH_2-His1 and ND1-His1; NE2-His28; NE2-His32) from the three histidines in all five models (Supplementary Table 4 and 5 and Supplementary Fig. 9) range between 1.4 and 3.5 Å, which is clearly infeasible when compared to the expected metal-ligand bond lengths of ca 2.0 Å. However, model 3 with Cu exhibited a range of metal-ligand bond lengths of 1.9 to 2.4 Å (Fig. 3c) which fall within the expected range. Moreover, the coordination geometry of the four ligands around the Cu ion, approximates to a square plane, consistent with that expected for Cu^{2+} coordination.

SUPPLEMENTARY FIGURES

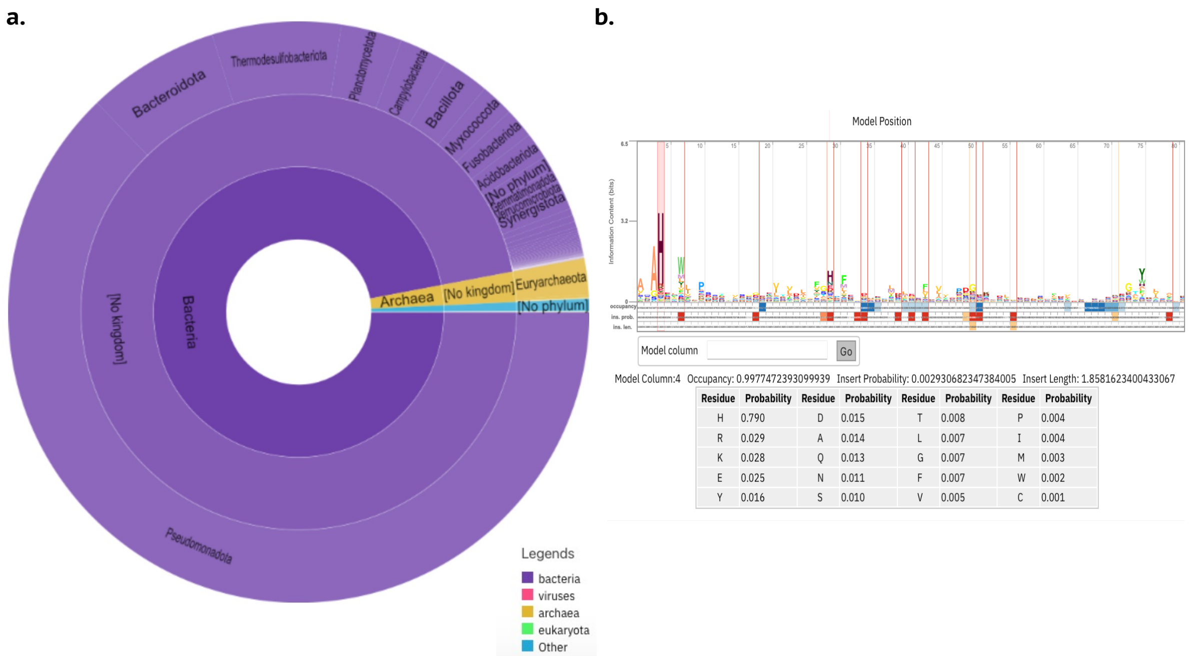

Supplementary Figure 1. DUF4198 Pfam domain features. **a.** Taxonomic distribution of DUF4198 genes from Pfam database, showing high abundance in the Pseudomonata phyla. **b.** DUF4198 domain signature from Pfam databse, showing the probability of occurrence of each amino acid in each motif positioning. Histidine is the N-terminal residue in 79% of the sequences.

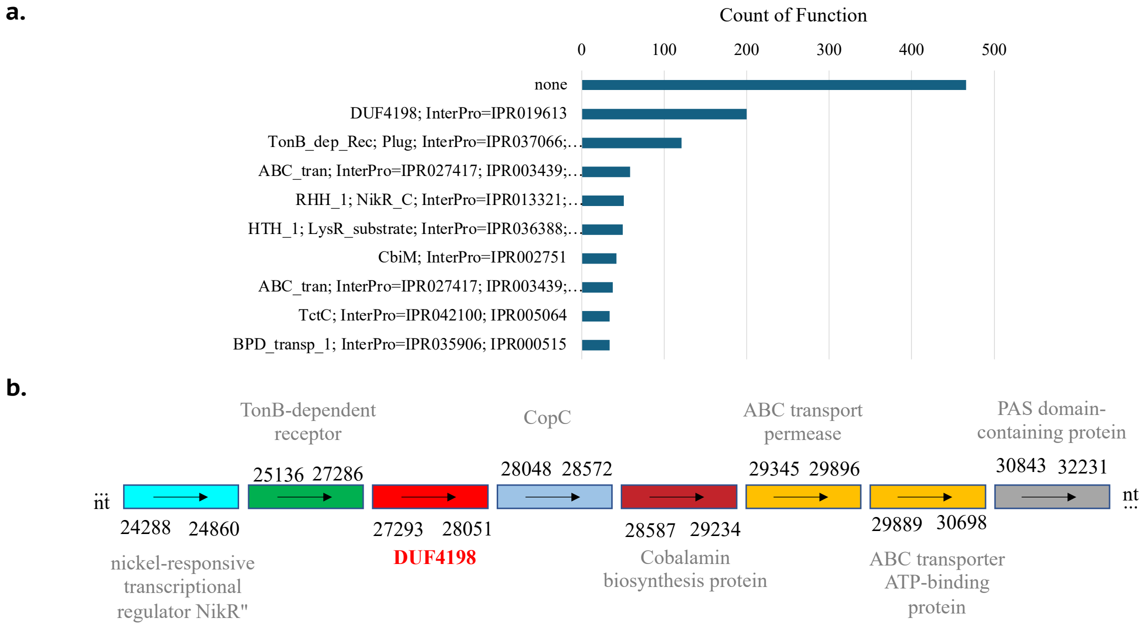

Supplementary Figure 2. DUF4198 genomic organization features. a. Counts of function for the DUF4198 neighbour genes across bacterial genomes from the RefSeq database. The protein families found in the neighbourhoods of DUF4198 containing ORFs were mostly of unknown function, TonB receptor proteins and those related to nickel-responsive transcriptional regulator NikR_C. This genome neighbourhood analysis relating DUF4198 to nickel biochemistry agrees with other clues obtained from transcriptomic studies, in which the domain was identified in components of the ATP-binding cassette transporters for nickel and cobalt, found in bacteria.[6] The proteins are also expressed in these organisms in response to iron-depletion. **b.** Organization of a DUF4198-containing operon in the genome of *I. sakaiensis*. Its neighbourhood genes are related to metal binding/transport. To the downstream region of *Is*DUF4198 there is a TonB receptor protein followed by transcriptional regulator NikR_C and upstream there is a CopC-like protein followed by cobalamin biosynthesis protein (vitamin B12). Source data are provided as a Source Data file.

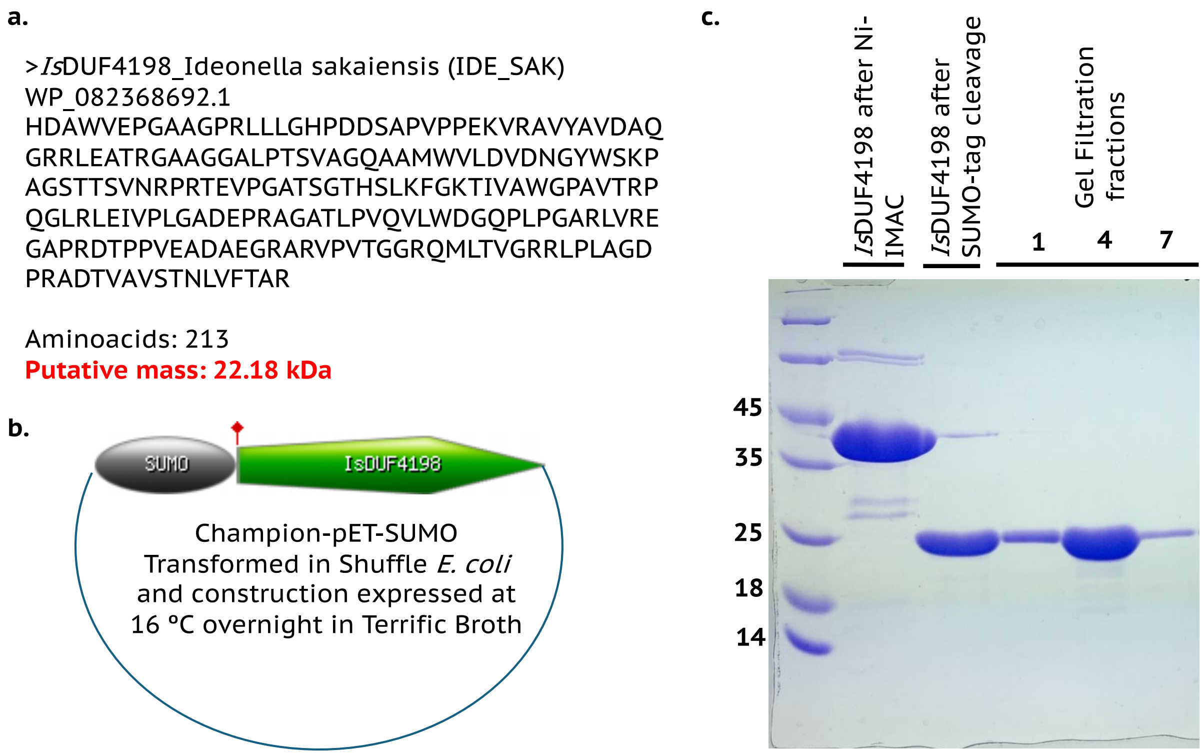

Supplementary Figure 3. Production of recombinant *Is*DUF4198. a. Amino acid sequence of expressed protein, produced from construct cloned without the signal peptide. **b.** Vector construct to produce recombinant *Is*DUF4198. **c.** 12% SDS-PAGE. Lane 1 – Ladder; Lane 2 – *Is*DUF4198 eluted from Ni-IMAC; Lane 3 – *Is*DUF4198 concentrated after SUMO-tag cleavage; Lanes 4-6 – Size exclusion fraction containing *Is*DUF4198.

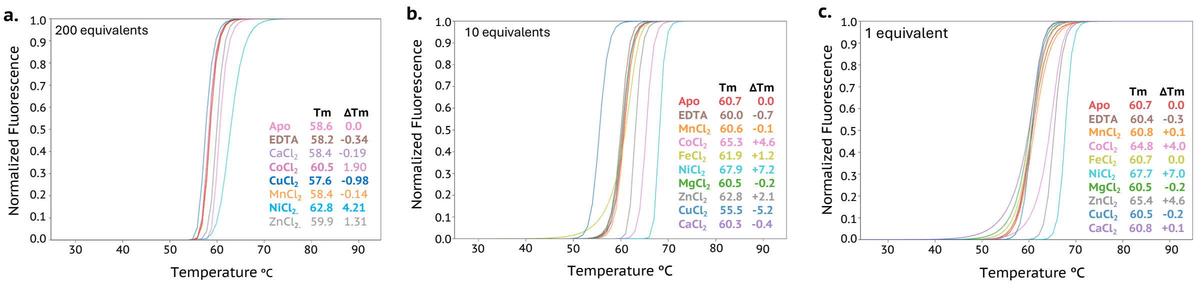

Supplementary Figure 4. Thermal shift analysis (TSA) of *Is*DUF4198 with several metals in different molar equivalent ratios. 30 µM *Is*DUF4198 with 200 molar equivalent of metals (**a**), 10 molar equivalents (**b**) and 1 molar equivalent (**c**). Each TSA curve is coloured according with its respective metal-Cl_2 as shown in the figure legend. Positive shifts indicate ligand binding to the proteins and thermal stabilization, while negative shifts indicate ligand binding to the proteins and thermal destabilization due to protein aggregation or precipitation. Source data are provided as a Source Data file.

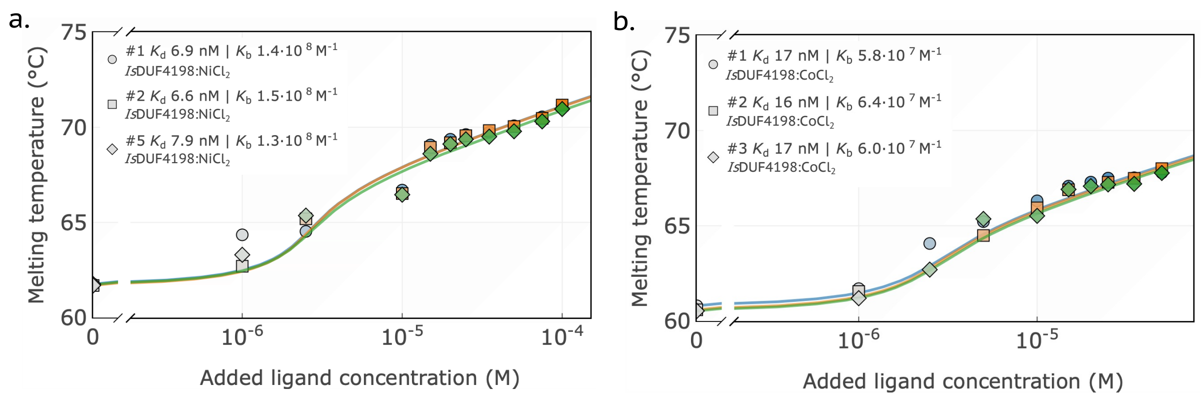

Supplementary Figure 5. Determination of dissociation constant (K_d) of *Is*DUF4198 in the presence of $NiCl_2$ (a) and $CoCl_2$ (b). Each replicate is shown as a different marker and colour: green (diamond), blue (circle) and orange (square). Lower and higher temperature of melting were also coloured in a colour gradient from brighter to darker. Source data are provided as a Source Data file.

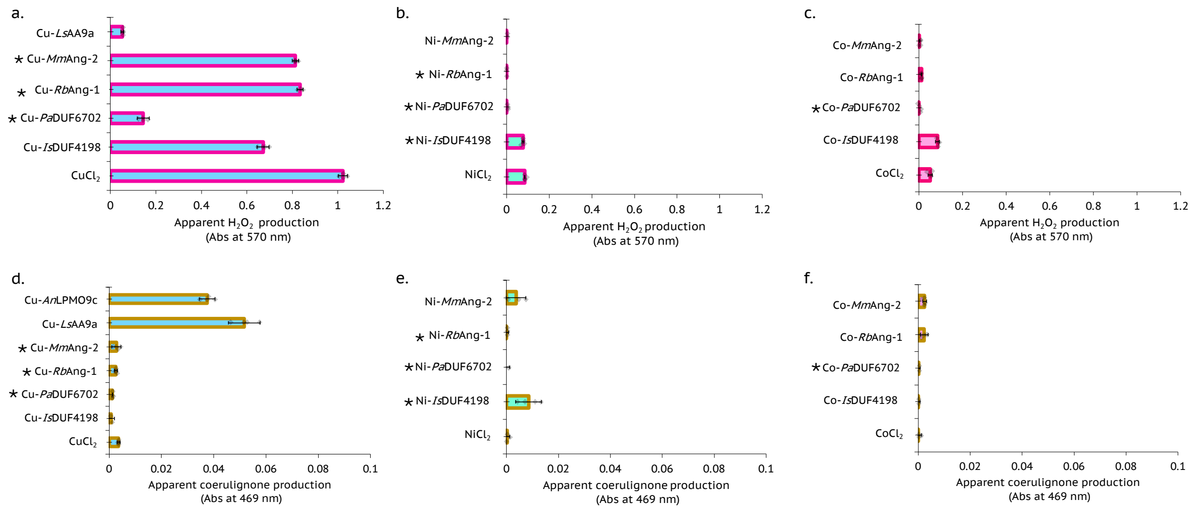

Supplementary Figure 6. Redox activities of the target proteins loaded with different metals. The oxidase activity of the target proteins loaded with copper (**a**), nickel (**b**) and cobalt (**c**) determined through the apparent hydrogen peroxide production in the presence of an electron donor (ascorbate). Note that LPMOs are known to only have very weak oxidase activities. The peroxidase activities of the target proteins loaded with copper (**d**), nickel (**e**) and cobalt (**f**) determined through the apparent coerulignone production derived from hydrogen peroxide consumption. Bar lines in all charts were coloured according to the colour of the reaction products of their respective enzyme assays (magenta for AmplexRed assay and brown to 2,6-dimethoxyphenol assay). Bars were hachured based on the metal colours of the target metal-loaded target proteins (copper: blue; nickel: green; cobalt: pink). *Indicates that metal binding studies associate the protein with tight binding of that metal ion. Source data are provided as a Source Data file.

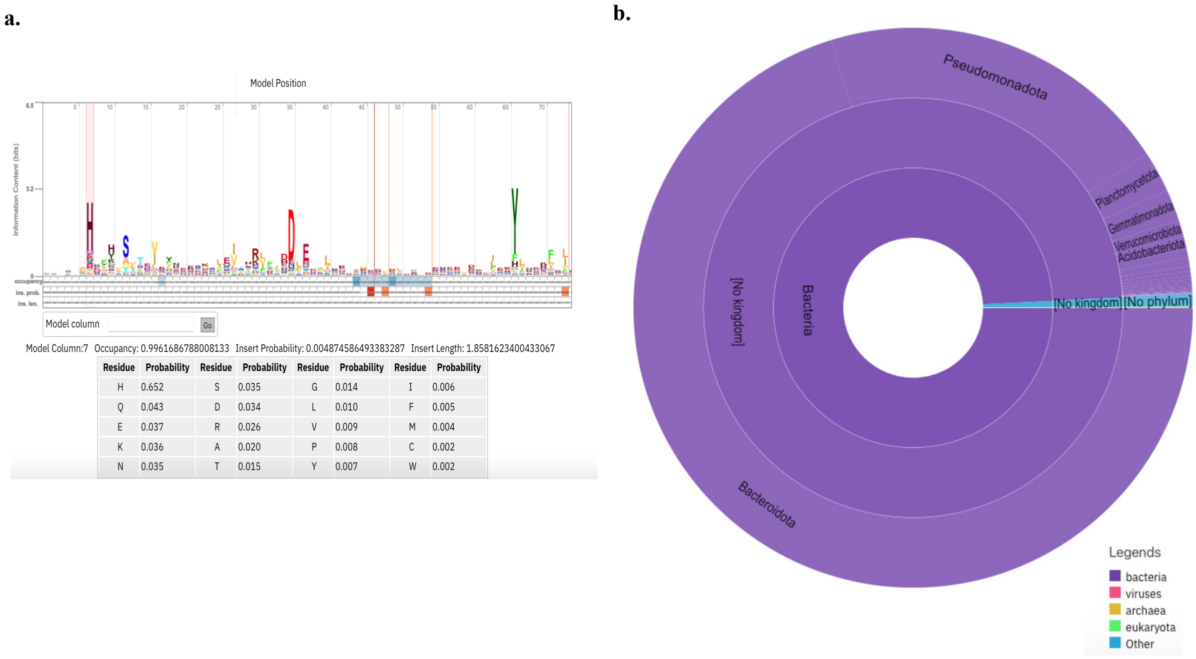

Supplementary Figure 7. DUF6702 Pfam domain features. **a.** DUF6702 domain signature from Pfam database, showing the probability of occurrence of each amino acid in each motif positioning. Histidine can be found as the N-terminal residue in 65% of the sequences. **b.** Taxonomic distribution of DUF6702 genes from Pfam database, showing high abundance in the Bacteroidota phylum.

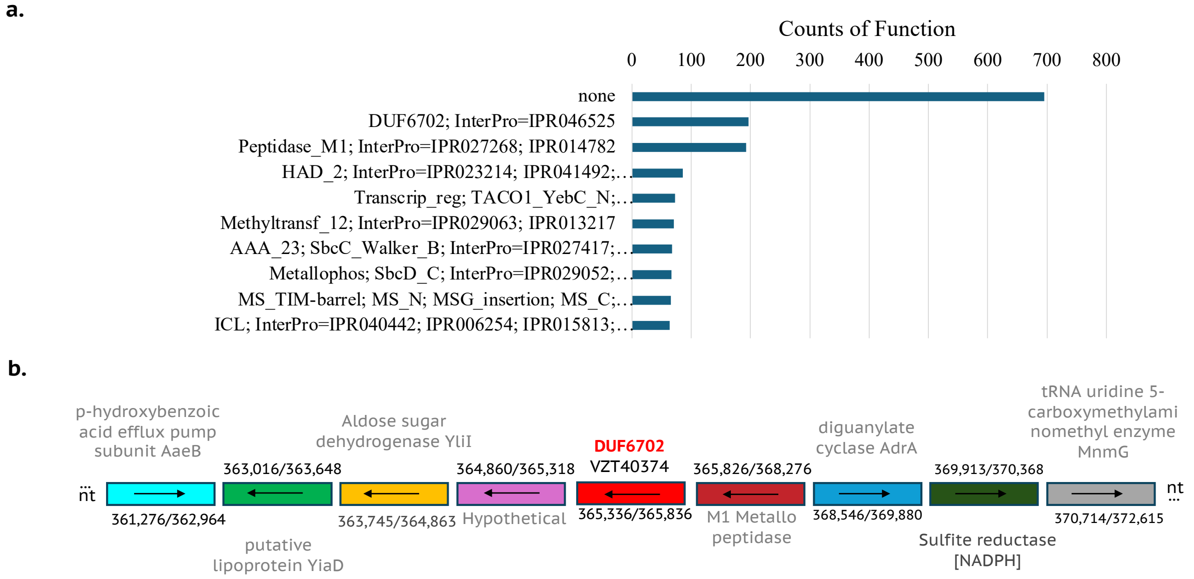

Supplementary Figure 8. DUF6702 genomic organization features. a. Counts of function for the DUF6702 neighbour genes across bacterial genomes from the RefSeq database. This analysis revealed that proteins with unknown functions were the most frequently encountered

neighbours of DUF6702. M1 peptidases and HAD_2 domain-containing proteins, predicted to be haloacid dehalogenase-like hydrolases, were also represented. M1 peptidases are a well-characterized group of enzymes, including the membrane-bound alanine aminopeptidase (EC 3.4.11.2). These enzymes belong to the HEXXH+E group and are found across diverse species. Notably, M1 peptidases utilize different metal ions—zinc, cobalt, manganese or copper—within their active sites.[7] The HAD superfamily encompasses a wide range of enzymes, including phosphatases, phosphonatases, P-type ATPases, beta-phosphoglucomutases, phosphomannomutases, and dehalogenases. Many HAD family enzymes also rely on metal ions as cofactors for their function.[8] The colocation of DUF6702 with M1 peptidases and HAD enzymes suggests that DUF6702 proteins could be involved in metal binding and possibly metal transport. **b.** Organization of a DUF6702-containing operon in the genome of *P. aeruginosa*. The neighbouring genes show at downstream of *Pa*DUF6702 is a hypothetical protein, followed by an aldose sugar dehydrogenase YliI. Upstream, an M1 peptidase is present, followed by the diguanylate cyclase AdrA. Source data are provided as a Source Data file.

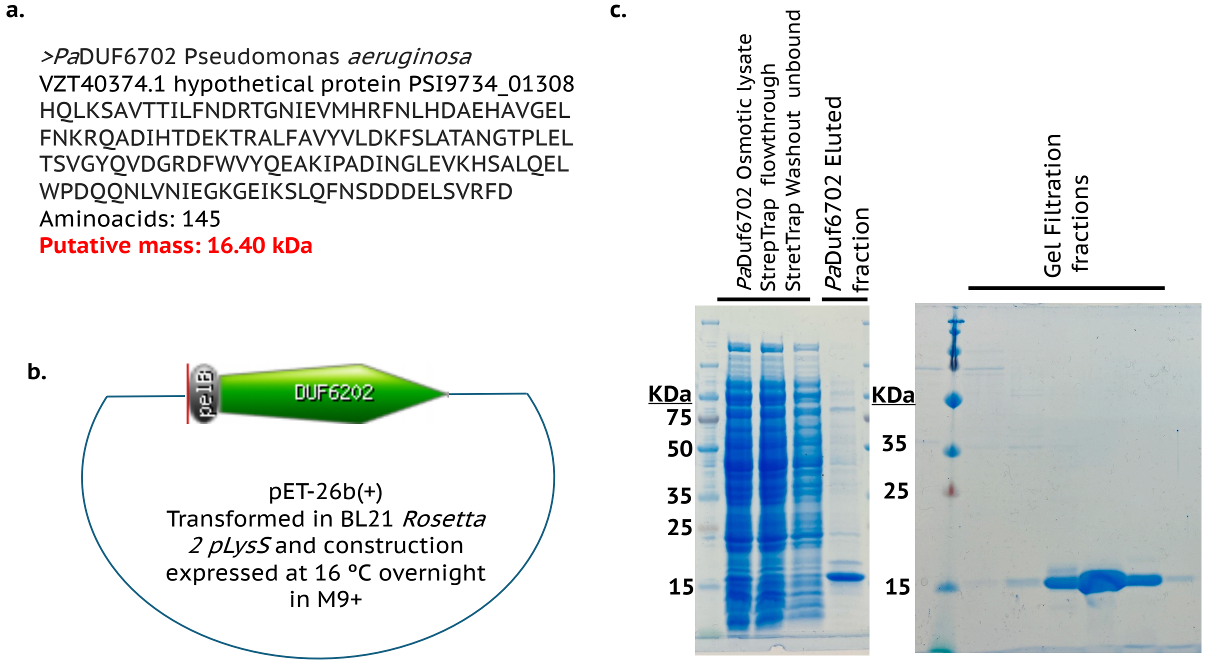

Supplementary Figure 9. Production of recombinant *Pa*DUF6702. a. cloned amino acid sequence lacking its original signal peptide. **b.** Vector construction to produce recombinant *Pa*DUF6702. **c. Gel 1:** 4-20% SDS-PAGE. Lane 1 – Ladder; Lane 2 – PaDUF6702 osmotic lysate; Lane 3 – Strep-Trap Flowthrough; Lane 4 - Strep-Trap washout unbound; Lane 5 fraction eluted from Strep-Trap with 50 mM biotin. **Gel 2:** 12% SDS-PAGE. Lane 1 – Ladder; Other lanes - Size exclusion fractions containing *Pa*DUF6702.

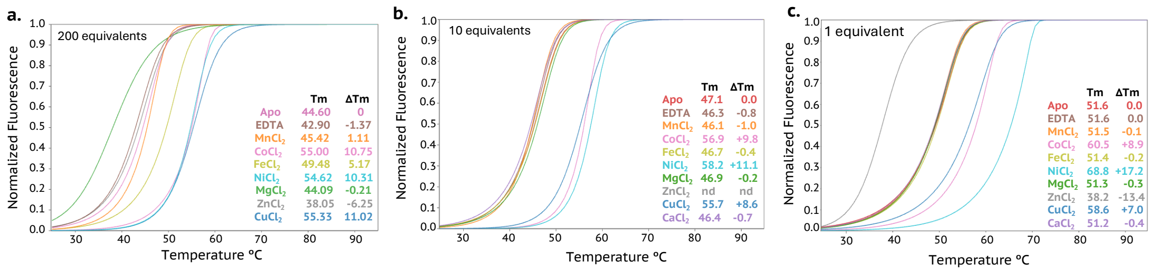

Supplementary Figure 10. Thermal shift analysis (TSA) of *Pa*DUF6702 with several metals in different molar equivalents. 30 µM *Pa*DUF4198 with 200 molar equivalent of metals (**a**), 10 molar equivalents (**b**) and 1 molar equivalent (**c**). Each TSA curve is coloured according with its respective metal-Cl_2 as shown in the figure legend. Positive shifts indicate ligand binding to the proteins and thermal stabilization, while negative shifts indicate ligand binding to the proteins and thermal destabilization due to protein aggregation or precipitation. Source data are provided as a Source Data file.

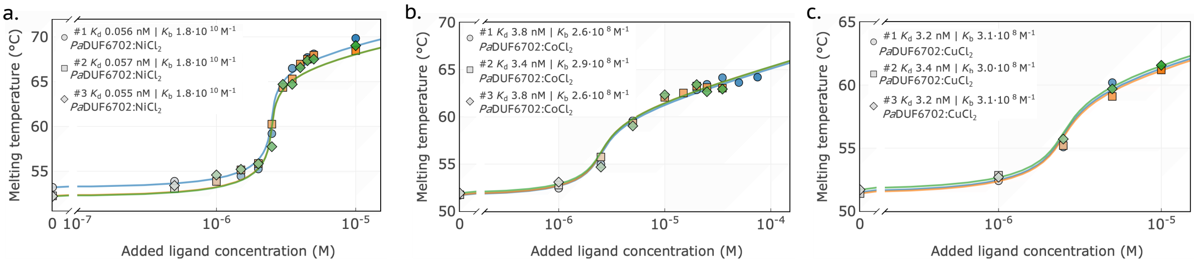

Supplementary Figure 11. Determination of dissociation constant (K_d) of *Pa*DUF6702 in the presence of $NiCl_2$ (a), $CoCl_2$ (b) and $CuCl_2$ (c). Each replicate is shown as a different marker and colour: green (diamond), blue (circle) and orange (square). Lower and higher temperature of melting were also coloured in a colour gradient from brighter to darker. Source data are provided as a Source Data file.

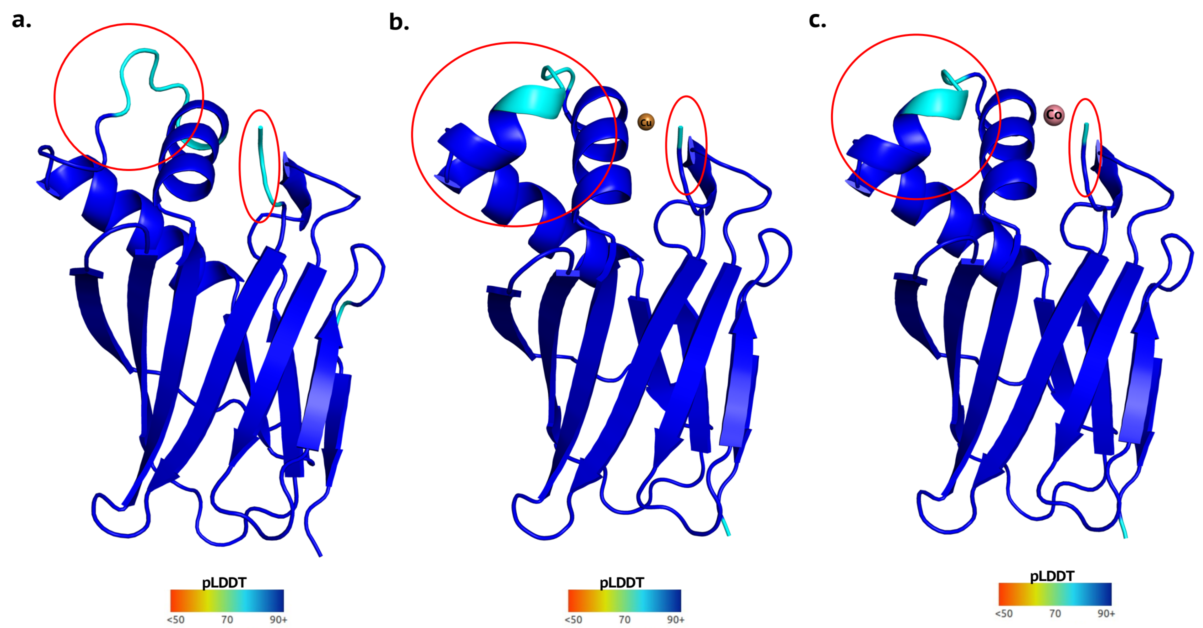

Supplementary Figure 12. AlphaFold 3 models of *Pa*DUF6702. Cartoon representation of *apo*- **(a)**, Cu- **(b),** and Co- **(b)** forms of *Pa*DUF6702, showing secondary structural elements coloured by pLDDT score. Ion modelling induced structural conformational changes in the Cu and Co bound structures highlighted in red circles.

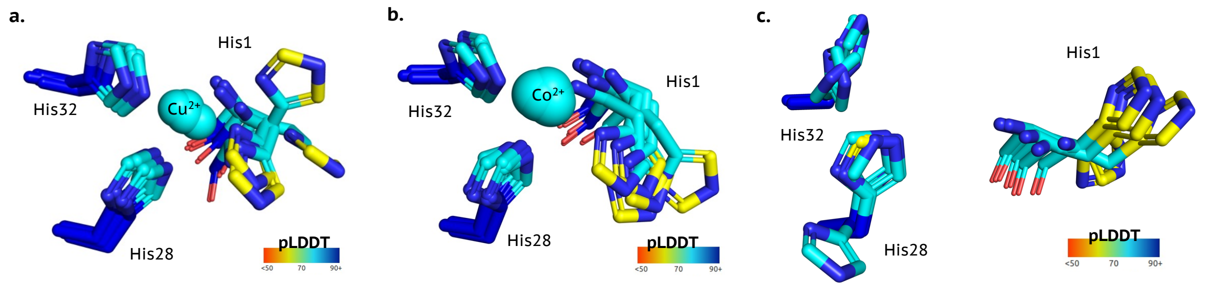

Supplementary Figure 13. Metal binding sites in the modelled structures of *Pa*DUF6702. Metal binding to the *Pa*DUF6702 model induced conformational changes for the side chains of the triple histidine brace. **a.** Detail of the Cu binding site showing a range of possible positions for the His1 side chain. **b.** Detail of the Co binding site showing showing a range of possible positions for the His1 side chain. **c.** Detail of the *apo* form binding site showing a range of possible positions for the the His1, His 28 and His32 side chains. The amino acids and metal centres were coloured according to their pLDDT score.

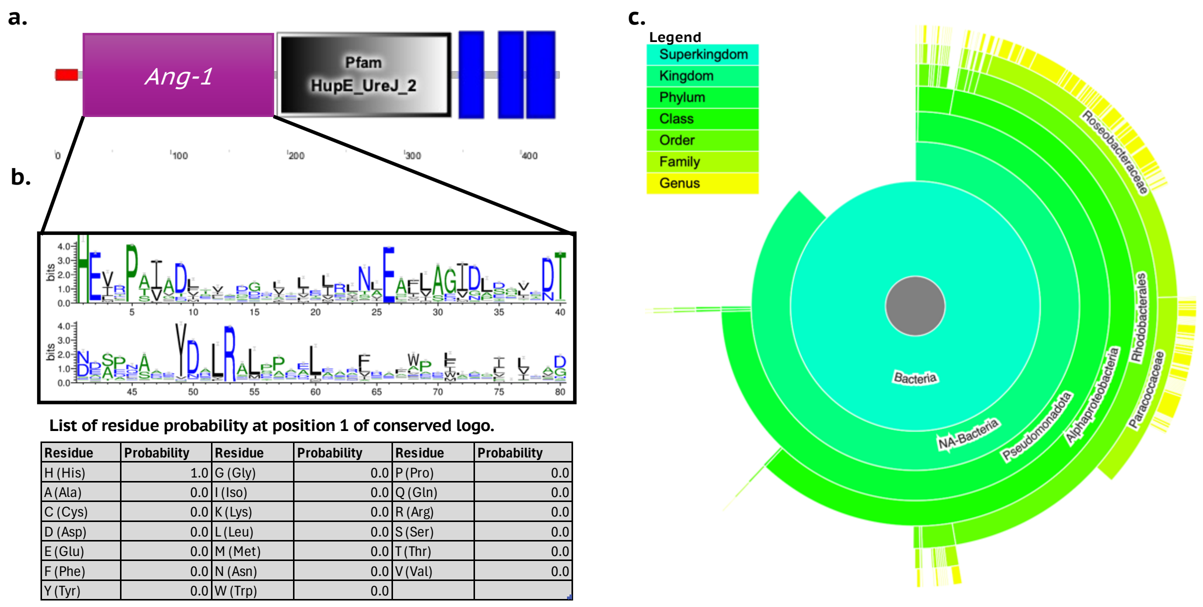

List of residue probability at position 1 of conserved logo.

Residue	Probability	Residue	Probability	Residue	Probability
H (His)	1.0	G (Gly)	0.0	P (Pro)	0.0
A (Ala)	0.0	I (Iso)	0.0	Q (Gln)	0.0
C (Cys)	0.0	K (Lys)	0.0	R (Arg)	0.0
D (Asp)	0.0	L (Leu)	0.0	S (Ser)	0.0
E (Glu)	0.0	M (Met)	0.0	T (Thr)	0.0
F (Phe)	0.0	N (Asn)	0.0	V (Val)	0.0
Y (Tyr)	0.0	W (Trp)	0.0		

Supplementary Figure 14. Ang-1 domain feature. a. Protein domain architecture generated in the SMART database. Red box is the signal peptide, purple box the putative new Ang-1 domain, black the Pfam HupE/UreJ-2 Pfam domain, and blue boxes are transmembrane regions. **b.** Ang-1 domain signature generated in ConservFold tool, showing the probability of occurrence of each amino acid in the position 1 of the domain motif. Histidine can be found as the N-terminal residue of 100% of the sequences. **c.** Taxonomic distribution of Ang-1 proteins, showing high counts in the Pseudomonadota phylum (generated on EFI-EST Database).

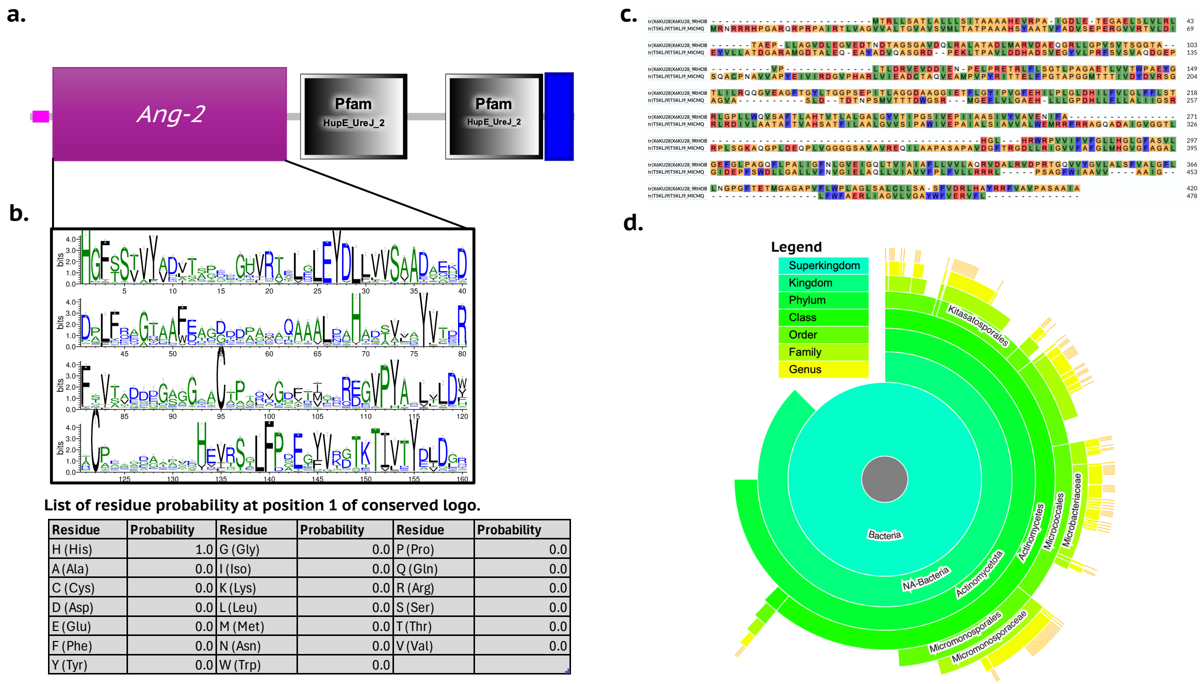

List of residue probability at position 1 of conserved logo.

Residue	Probability	Residue	Probability	Residue	Probability
H (His)	1.0	G (Gly)	0.0	P (Pro)	0.0
A (Ala)	0.0	I (Iso)	0.0	Q (Gln)	0.0
C (Cys)	0.0	K (Lys)	0.0	R (Arg)	0.0
D (Asp)	0.0	L (Leu)	0.0	S (Ser)	0.0
E (Glu)	0.0	M (Met)	0.0	T (Thr)	0.0
F (Phe)	0.0	N (Asn)	0.0	V (Val)	0.0
Y (Tyr)	0.0	W (Trp)	0.0		

Supplementary Figure 15. Ang-2 domain feature. **a.** Protein domain architecture generated in the SMART database. Pink box is the signal peptide, purple box the putative new Ang-2

domain, black the Pfam HupE/UreJ-2 Pfam domain, and blue boxes are transmembrane regions. **b.** Ang-2 domain signature generated in ConservFold tool, showing the probability of occurrence of each amino acid in the position 1 of the domain motif. Histidine can be found as the N-terminal residue of 100% of the sequences. **c.** Alignment on Clustal-Uniprot between the full-length sequences of *Rb*Ang-1a-HupE/UreJ-2 and *Mm*Ang-2a-HupE/UreJ-2 (Only the Ang domains alignment is displayed). **d.** Taxonomic distribution of Ang-2 proteins, showing high counts in the Actinomycetota phylum (generated on EFI-EST Database).

a.

>*Rb*Ang-1a_X6KU28_Membrane protein
OS=Rhodobacteraceae bacterium PD-2 -
P279_24175 ETA49561.1
HEVRPAIGDLETEGAELSLVLRLTAEPLLAGVDLEGVEDT
NDTAGSGAVDQLRALATADLMARVDAEQGRLLGPVSV
TSGGTAVPLTLDRVEVDDIENPELPRETRLFLSGTLPAG
AETLVVTWPAEYGTLILRQQGVEAGFTGYLTGGPSEPIT
LAGGDA
Aminoacids: 169
Putative mass: 17.7 kDa

b.

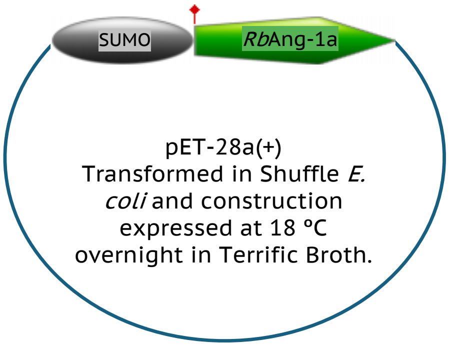

c.

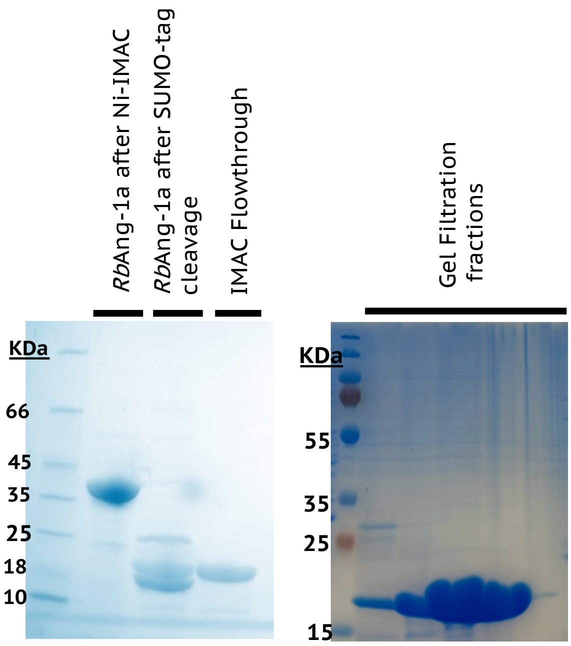

Supplementary Figure 16. Production of recombinant *Rb*Ang-1a. Amino acid sequence of expressed protein, produced from construct cloned without the signal peptide. **b.** Vector construct to produce recombinant *Rb*Ang-1a. **c. Gel 1:** 12% SDS-PAGE. Lane 1 – Ladder; Lane 2 – *Rb*Ang-1a eluted from 1st Ni-IMAC; Lane 3 – *Rb*Ang-1a concentrated after SUMO-tag cleavage; Lane 4 – 2nd Immobilized metal affinity chromatography (IMAC) flowthrough. **Gel 2:** 12% SDS-PAGE. Lane 1 – Ladder; Other lanes - Size exclusion fractions containing *Rb*Ang-1a.

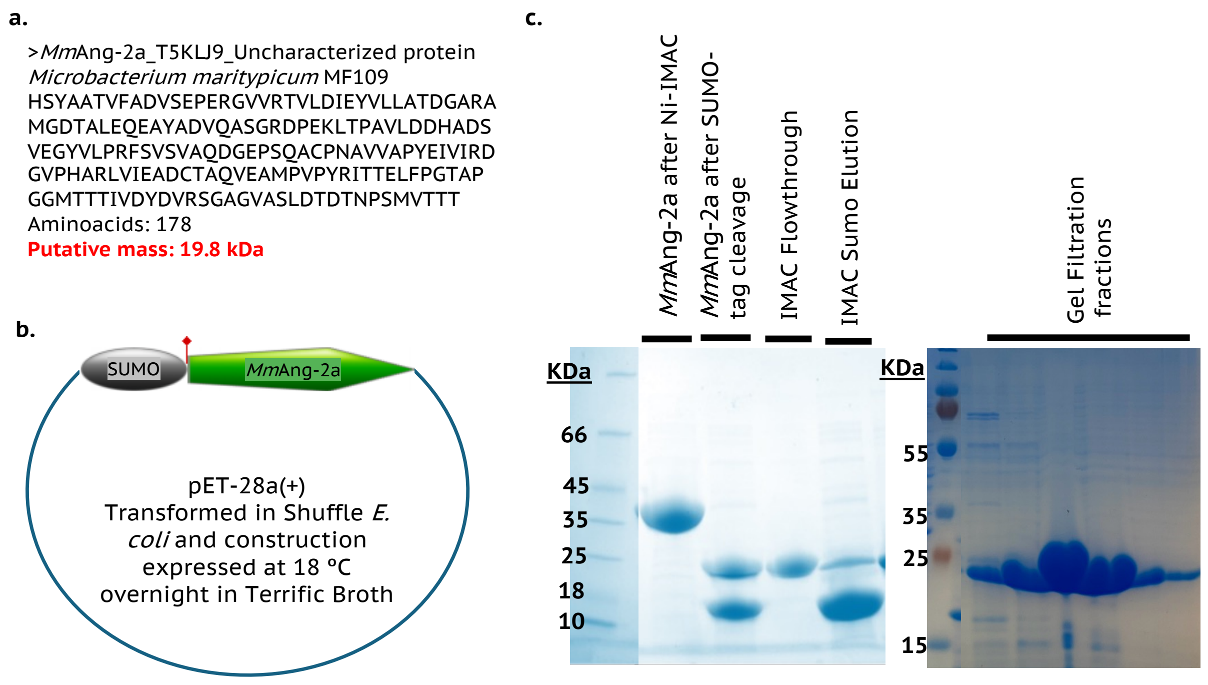

Supplementary Figure 17. Production of recombinant *Mm*Ang-2a. Amino acid sequence of expressed protein, produced from construct cloned without the signal peptide. **b.** Vector construct to produce recombinant *Mm*Ang-2a. **c. Gel 1:** 12% SDS-PAGE. Lane 1 – Ladder; Lane 2 –*Mm*Ang-2a eluted from 1st Ni-IMAC; Lane 3 – *Mm*Ang-2a concentrated after SUMO-tag cleavage; Lane 4 – 2nd IMAC flowthrough. **Gel 2:** 12% SDS-PAGE. Lane 1 – Ladder; Other lanes - Size exclusion fractions containing *Mm*Ang-2a.

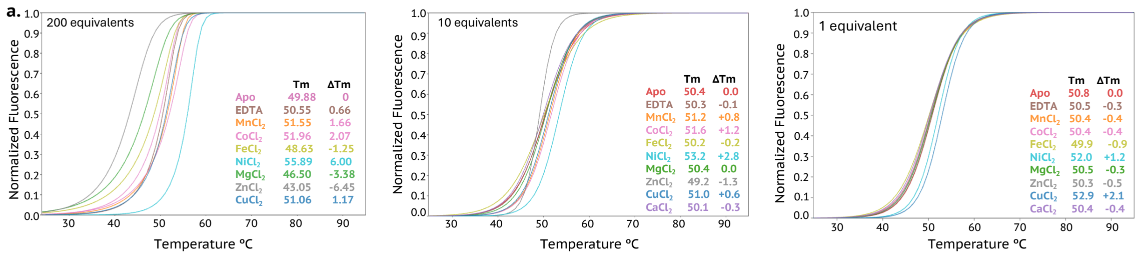

Supplementary Figure 18. Thermal shift analysis (TSA) of *Rb*Ang-1a with several metals in different molar equivalents ratios. 30 µM *Rb*Ang-1a with 200 molar equivalent of metals (**a**), 10 molar equivalents (**b**) and 1 molar equivalent (**c**). Each TSA curve is coloured according with its respective metal-Cl_2 as shown in the figure legend. Positive shifts indicate ligand binding to the proteins and thermal stabilization, while negative shifts indicate ligand binding to the proteins and thermal destabilization due to protein aggregation or precipitation. Source data are provided as a Source Data file.

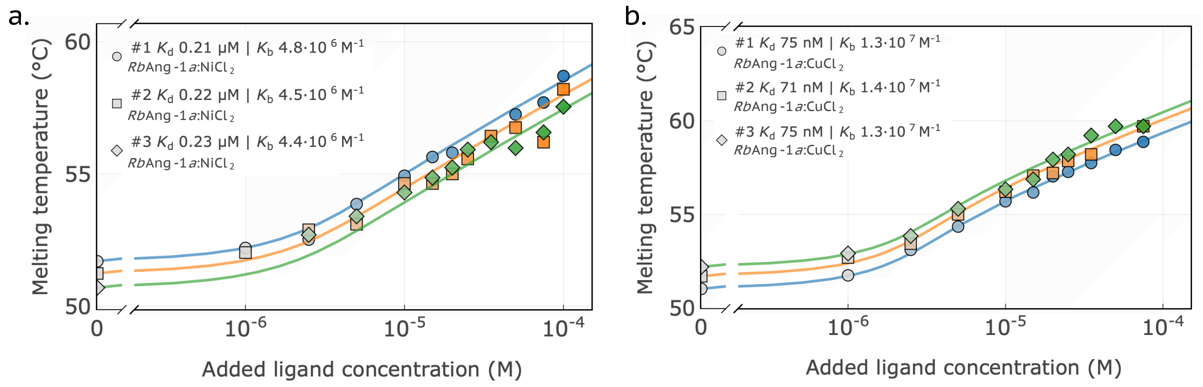

Supplementary Figure 19. Determination of dissociation constant (K_d) of *Rb*Ang-1a in the presence of $NiCl_2$ (a) and $CuCl_2$ (b). Each replicate is shown as a different marker and colour: green (diamond), blue (circle) and orange (square). Lower and higher temperature of melting were also coloured in a colour gradient from brighter to darker. Source data are provided as a Source Data file.

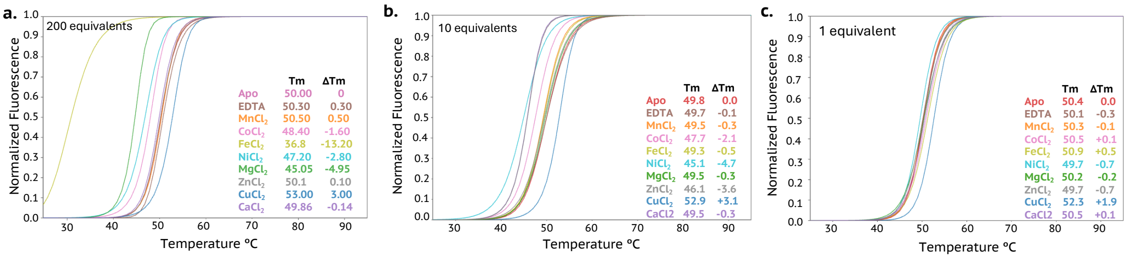

Supplementary Figure 20. Thermal shift analysis (TSA) of *Mm*Ang-2a with several metals in different molar equivalents ratios. 30 μM *Mm*Ang-2a with 200 molar equivalent of metals (**a**), 10 molar equivalents (**b**) and 1 molar equivalent (**c**). Each TSA curve is coloured according with its respective metal-Cl_2 as shown in the figure legend. Positive shifts indicate ligand binding to the proteins and thermal stabilization, while negative shifts indicate ligand binding to the proteins and thermal destabilization due to protein aggregation or precipitation. Source data are provided as a Source Data file.

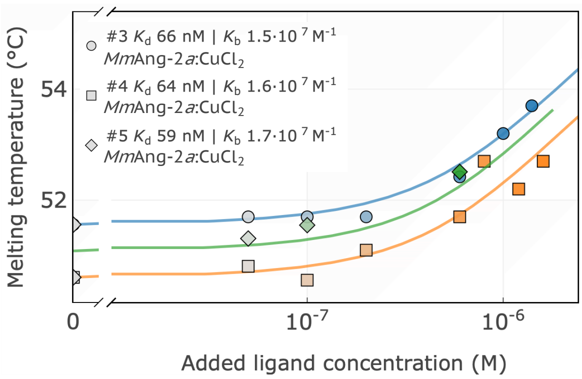

Supplementary Figure 21. Determination of dissociation constant (K_d) of *Mm*Ang-2a in the presence of $CuCl_2$. Each replicate is shown as a different marker and colour: green (diamond), blue (circle) and orange (square). Lower and higher temperature of melting were also coloured in a colour gradient from brighter to darker. Source data are provided as a Source Data file.

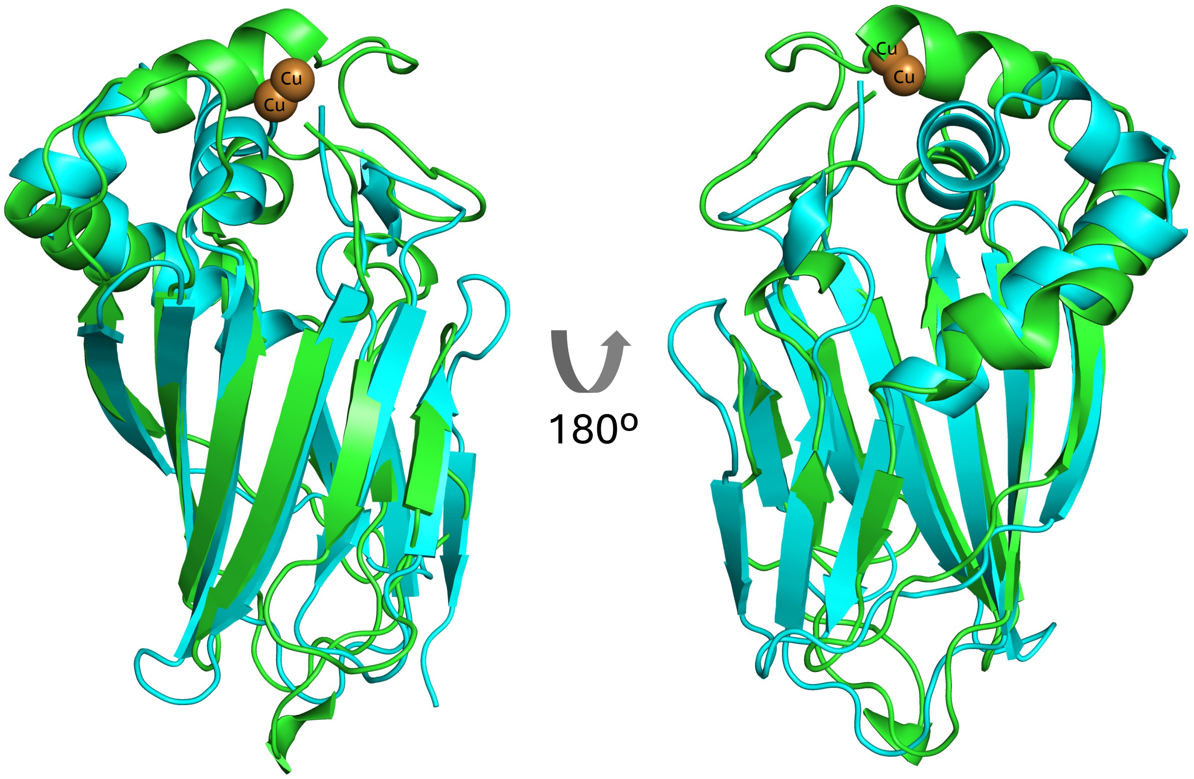

Supplementary Figure 22. Overlay of Cu-*Rb*Ang-1a structure (green) with the predicted model of Cu-*Pa*DUF6702 (cyan). *Rb*Ang-1a resembles *Pa*DUF6702 in fold, indeed, the alignment between the two structures generated a RMSD of 6.66 Å. Cu^+ centre is in brown.

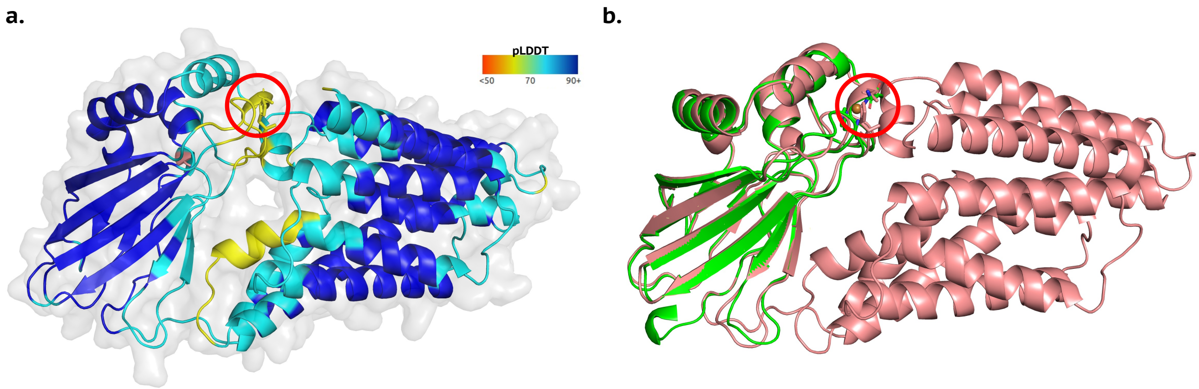

Supplementary Figure 23. Ribbon diagram to show overall model features of the full length *Rb*Ang-1a. a. The AlphaFold 3 (AF3) model of full length *Rb*Ang-1a-HupE/UreJ-2, showing secondary structural elements coloured by pLDDT scores; Van-der-Waals surface (light grey). **b.** Overlay of Cu-*Rb*Ang-1a structure (green) with the predicted model of full-length *Apo Rb*Ang-1a (salmon). *Rb*Ang-1a's metal binding site is highlighted in red circles.

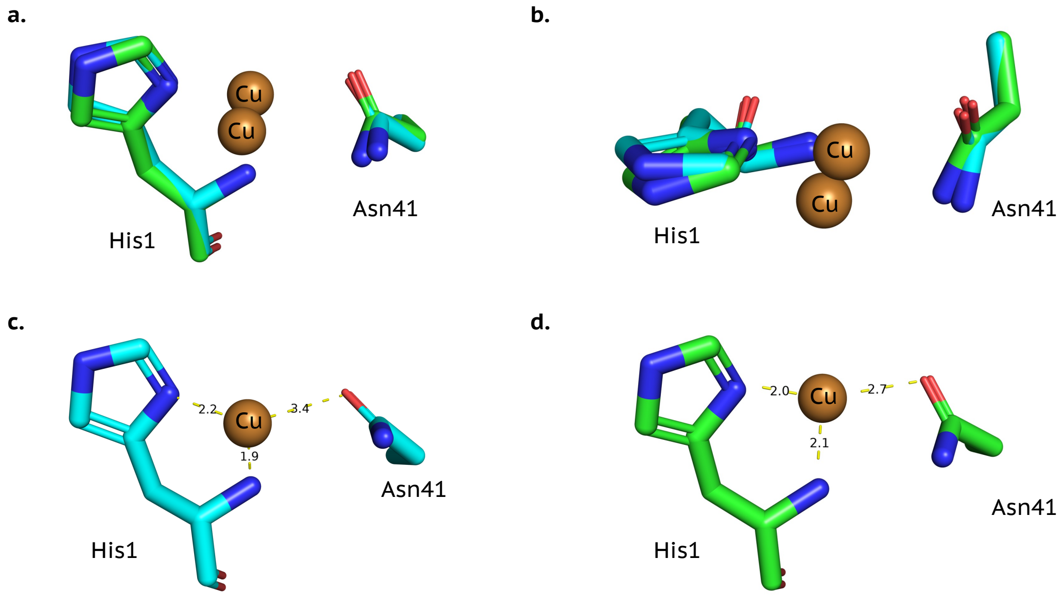

Supplementary Figure 24. Detail of metal binding sites of the AlphaFold 3 and crystal structure of *Rb*Ang-1a. a. Overlay of the copper binding site for the AF3 model (cyan) and crystal structure (green) of *Rb*Ang-1a (His1 and Asn41). **b.** Rotated version of overlay in **a**. **c.** Histidine brace Cu coordination distances for AF3 model of *Rb*Ang-1a. **d.** Cu coordination distances in *Rb*Ang-1a crystal structure. Cu^{+} centre is in brown.

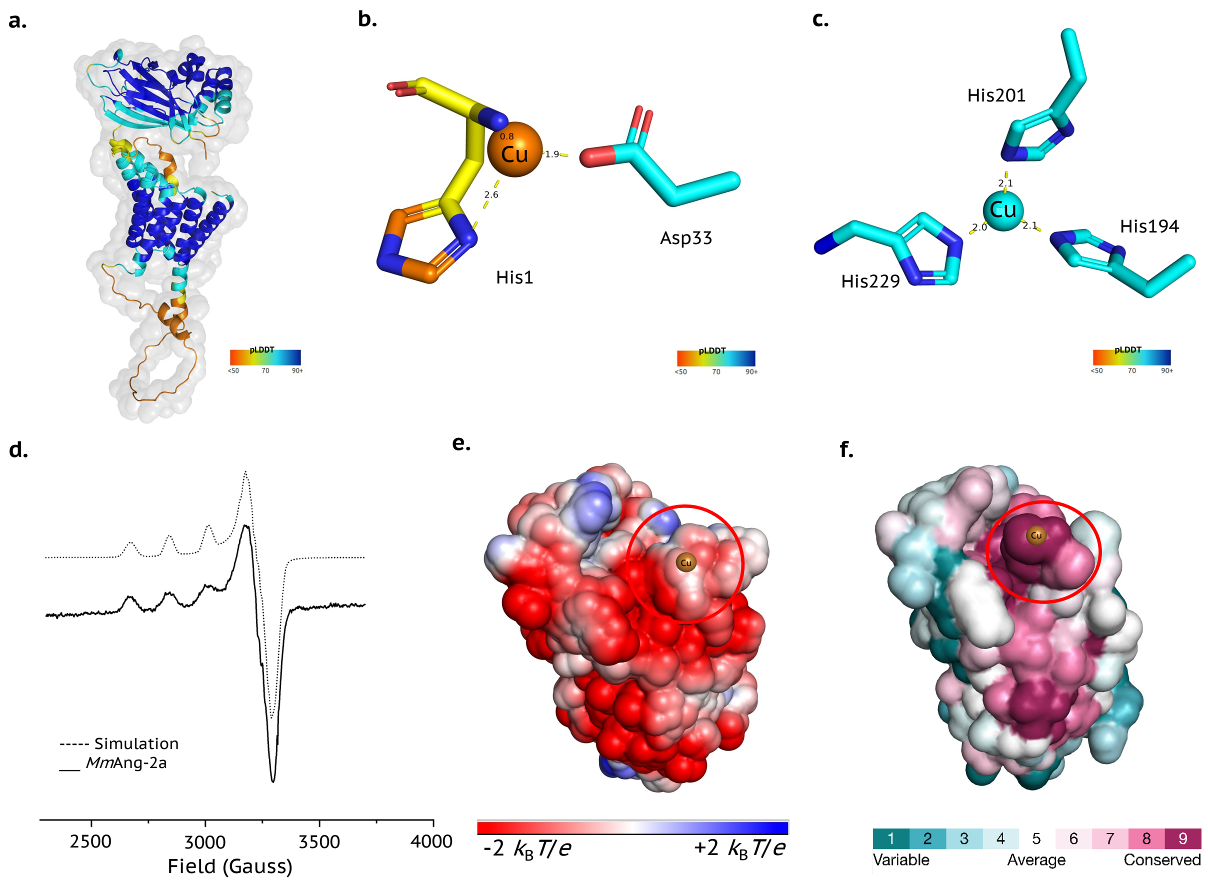

Supplementary Figure 25. Structural diagrams of Cu-containing Ang-2-HupE/UreJ-2. **a.** Ribbon diagram of AF3 model of *apo* form *Mm*Ang-2a-HupE/UreJ-2 showing secondary structural elements, Van-der-Waals surface (light grey) and amino acid residues molecules coloured by pLDDT scores; **b.** Detail of the metal-binding site of *Mm*Ang-2a. Cu^+ centre is in brown; **c.** Detail of the metal-binding site of the HupE/UreJ-2 domain of *Mm*Ang-2a-HupE/UreJ-2. Cu^+ centre is in blue showing its pLDTT score; Distances in angstroms among the Cu^{1+} centre and neighbouring His1 N atoms and Asn41 O atom. **d.** X-band, CW-EPR spectrum (150 K) of Cu^{2+} loaded form of *Mm*Ang-2a. **e.** Electrostatic surface potential of Cu loaded *Mm*Ang-2a generated by the APBS plugin for PyMol settled at pH 7.0 to calculate and visualize the surface electrostatic potential at ±2 k_BT/e. Cu^+ centre is in brown. **f.** Consurf sequence conservation analysis of *Apo Mm*Ang-2a. Cu^+ centre is in brown. Source data are provided as a Source Data file.

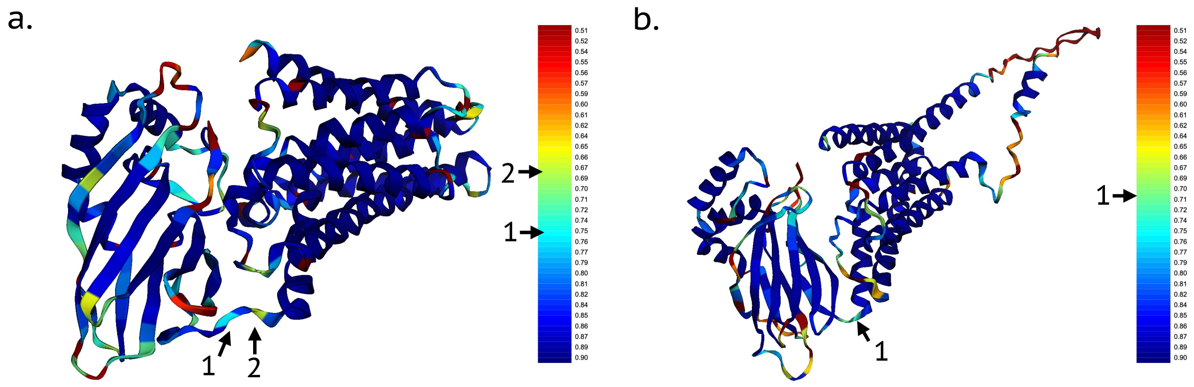

Supplementary Figure 26. Protein flexibility analysis of the Anglerases. The full-length AF3 models of *Rb*Ang-1a-HupE/UreJ-2 (**a**) and *Mm*Ang-2a-HupE/UreJ-2 (**b**) were submitted to flexibility analysis to determine the S^2 order parameters for backbone N−H vectors. Red means low S^2 score and highly disordered region and therefore high flexibility and dark "navy" blue high S^2 score and low disordered region and therefore no flexibility. The analysis for both *Anglerases* showed medium flexibility in the linker region between the Anglerase domain and the HupE/UreJ-2 domain.

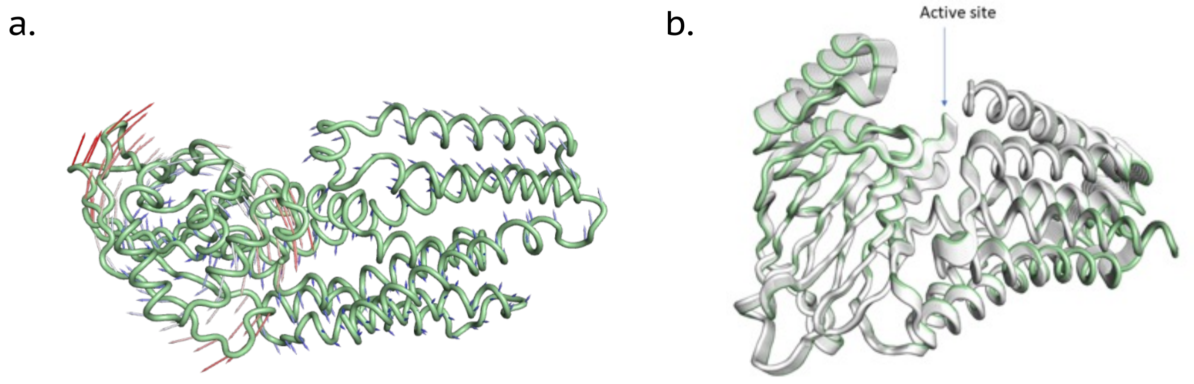

Supplementary Figure 27. Interdomain dynamic simulations of protein motion for the Anglerases. The simulations were performed using normal mode analyses. The vector diagram (largest displacement shown as red arrows which predicts relative protein motion) unambiguously shows the high relative dynamics of the anglerase domain *Rb*Ang-1a to that of the HupE/UreJ-2 domain. It also shows that the Ang1 domain rotates away from the HupE/UreJ-2 domain and, in doing so, exposes the metal binding site to solution (**a**). The results of the protein motion and dynamics analysis are less clear cut than that for Ang-1, as the AlphaFold structure of the *Mm*Ang-2a-HupE/UreJ-2 (**b**) complex contains a large, disordered alpha-helical loop (not shown) which dominates the relative sizes of predicted protein motion.

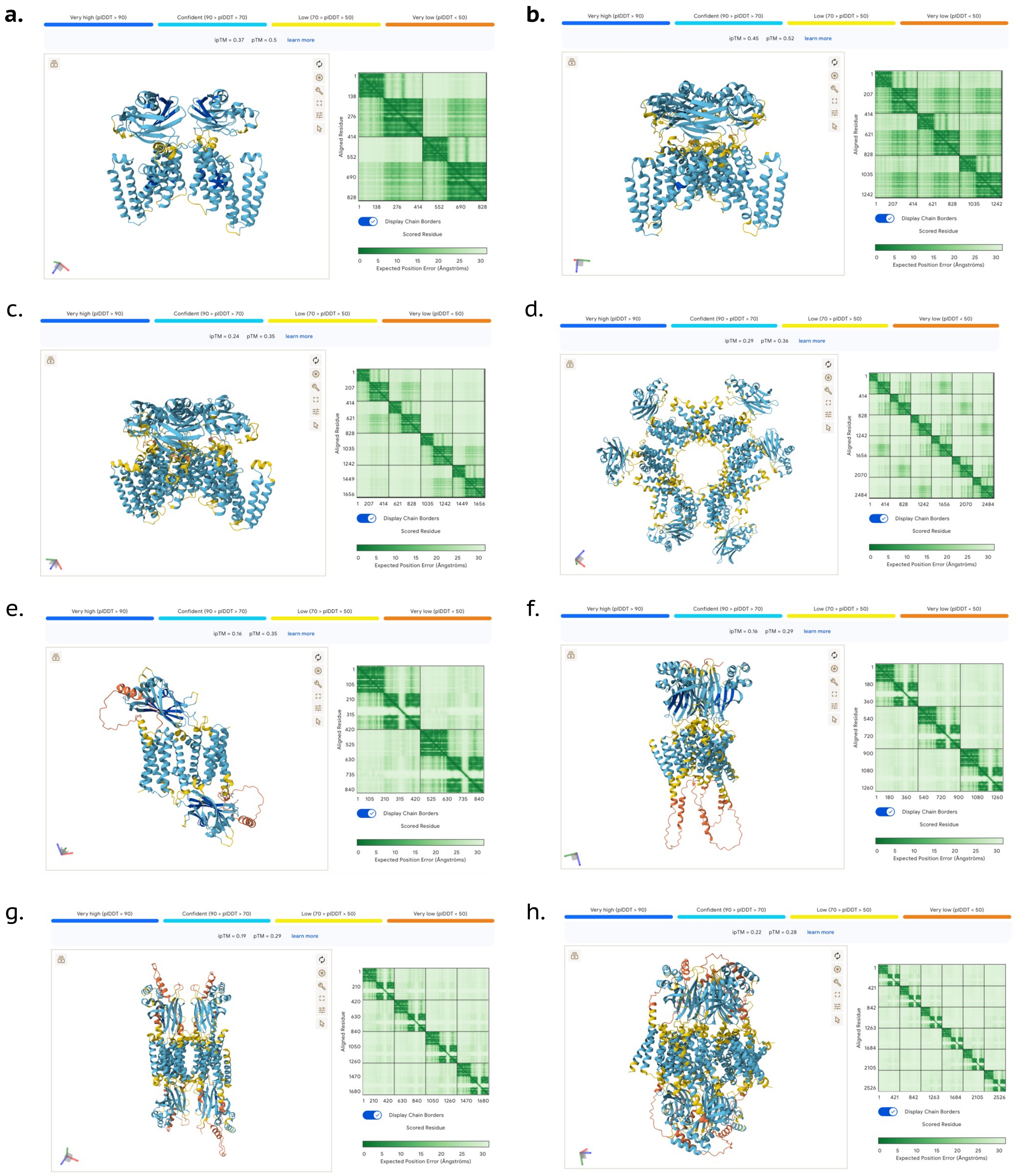

Supplementary Figure 28. Oligomeric state predictions for the Anglerases. AF3 predictions for the dimer **(a)**, trimer **(b)**, tetramer **(c)** and hexamer **(d)** states of the full-length protein sequences of *Rb*Ang-1a-HupE/UreJ-2 and dimer **(f)**, trimer **(e)**, tetramer **(g)** and hexamer **(h)** states of the full-length protein sequences of *Mm*Ang-2a-HupE/UreJ-2. Both the ipTM and pTM scores lower than 0.5 suggest low likelihoods of homo-multimeric complexes.

SUPPLEMENTARY TABLES

Supplementary Table 1. X-ray Data and refinement statistics for *Is*DUF4198

Data collection	
Space group	*P*1
Cell dimensions	
a, *b*, *c* (Å)	43.5, 47.5, 49.4
α, β, γ (°)	81.4, 81.5, 83.6
Resolution (Å)	48.46 - 1.44 (1.47 - 1.44)
R_{merge}	0.04 (0.25)
R_{pim}	0.02 (0.25)
$CC_{1/2}$	0.998 (0.909)
I / □*I*	18.2 (1.9)
Completeness (%)	64.1 (4.1)*
Redundancy	3.3 (1.2)
Refinement	
No. reflections	44705
R_{work} / R_{free}	0.13/0.18
No. atoms	
Protein	3323
Ligand/ion	2
Water	482
B-factors	
Protein	14.1
Ligand/ion	23.1
Water	26.1
R.m.s deviations	
Bond lengths (Å)	0.009
Bond angles (°)	1.733
Ramachandran plot residues	98.3
In most favourable regions (%)	1.7
In allowed regions (%)	0.0
PDB code	9GCB

* Low completeness reflects integration into the corners of a square detector. The data are 97.2% complete (90.2% in outer shell) to 1.8 Å.

Supplementary Table 2. List of bond distances and angles at nickel binding site of DUF4198.

Bond lengths	/A (esd ~ 0.1 Å)	Bond angles	/° (esd ~ 2°)
Ni-NH_2	2.3, 2.2*	NH_2-Ni-N(His1)	92, 90
Ni-N(His1)	2.1, 2.1	NH_2-Ni-N(His 18)	94, 94
Ni-N(His18)	2.1, 2.1	NH_2-Ni-O1 MLI	84, 93
Ni-O1 MLI (*trans* to NH_2)	2.0, 2.0	NH_2-Ni-O2 MLI	174, 169
Ni-O2 MLI (*trans* to His1)	2.1, 2.1	NH_2-Ni-O1 Water	88, 85
Ni-O1 Water (*trans* to NH_2)	2.0, 2.1	N(His1)-Ni-N(His18)	97, 96
		N(His1)-Ni-O1 MLI	175, 173
		N(His1)-Ni-O2 MLI	89, 96
		N(His1)-Ni-O1 Water	85, 86
		N(His18)-Ni-O1 MLI	86, 87
		N(His18)-Ni-O2 MLI	90, 92
		N(His18)-Ni-O1 Water	175, 177
		O1 MLI-Ni-O2 MLI	93, 78
		O1 MLI-Ni-O1 Water	86, 89
		MLI O2-Ni-O1 Water	91, 86

* Values are given for Ni in molecule A and molecule B respectively.

Supplementary Table 3. Global pLDDT values for *Pa*DUF6702 models.

Model	Model 0 pLDDT	Model 1 pLDDT	Model 2 pLDDT	Model 3 pLDDT	Model 4 pLDDT
apo *Pa*DUF6702	92.66	92.61	92.59	92.53	91.68
Cu modelled *Pa*DUF6702	93.96	92.66	93.05	94.19	93.43
Co modelled *Pa*DUF6702	94.85	93.27	93.95	93.15	93.08

Supplementary Table 4. Bond distances at cobalt binding site in *Pa*DUF6702 models, and ion model scores.

Metal-Nitrogen	Model 0 bond distance	Model 1 bond distance	Model 2 bond distance	Model 3 bond distance	Model 4 bond distance
Co-NH_2 (His1)	1.8 Å	2.1 Å	1.7 Å	2.5 Å	1.4 Å
Co-N (His 1)	3.2 Å	2.8 Å	3.7 Å	5.3 Å	4.0 Å
Co-N (His 28)	2.2 Å	2.2 Å	2.2 Å	2.2 Å	2.1 Å
Co-N (His 32)	2.5 Å	2.5 Å	2.5 Å	2.4 Å	2.4 Å

	Model 0 Scores	Model 1 Scores	Model 2 Scores	Model 3 Scores	Model 4 Scores
ipTM	0.90	0.89	0.87	0.86	0.86
pTM	0.93	0.92	0.92	0.91	0.91

Supplementary Table 5. Bond distances at copper binding site in *Pa*DUF6702 models, and ion model scores.

Metal-Nitrogen	Model 0 bond distance	Model 1 bond distance	Model 2 bond distance	Model 3 bond distance	Model 4 bond distance
Cu-NH_2 (His1)	2.0 Å	2.3 Å	2.5 Å	1.9 Å	1.8 Å
Cu-N (His 1)	2.6 Å	2.4 Å	3.1 Å	2.4 Å	3.5 Å
Cu-N (His 28)	2.1 Å	2.1 Å	2.1 Å	2.1 Å	2.1 Å
Cu-N (His 32)	2.4 Å	2.3 Å	2.3 Å	2.3 Å	2.4 Å
	Model 0 Scores	Model 1 Scores	Model 2 Scores	Model 3 Scores	Model 4 Scores
ipTM	0.92	0.91	0.91	0.92	0.91
pTM	0.92	0.91	0.91	0.90	0.89

Supplementary Table 6. Data collection and refinement statistics for *Rb*Ang-1a.

Data collection	
Space group	$P3_221$
Cell dimensions	
a, *b*, *c* (Å)	79.6, 79.6, 63.4
α, β, γ (°)	90.0, 90.0. 120.0
Resolution (Å)	46.68 - 2.25 (2.32 - 2.25)
R_{merge}	0.14 (1.67)
R_{pim}	0.03 (0.43)
$CC_{1/2}$	0.995 (0.618)
I / □*I*	12.8 (2.0)
Completeness (%)	100.0 (100.0)
Redundancy	17.7 (16.2)
Refinement	
No. reflections	11371
R_{work} / R_{free}	0.22/0.25
No. atoms	1245
Protein	1210
Ligand/ion	4
Water	31

B-factors	
Protein	62.0
Ligand/ion	86.5
Water	55.6
R.m.s deviations	
Bond lengths (Å)	0.014
Bond angles (º)	2.372
Ramachandran plot residues	98.8
In most favorable regions (%)	1.2
In allowed regions (%)	0.0
PDB code	9GCE

Supplementary Table 7. List of bond distances and angles at nickel binding site of *Rb*Ang-1a.

Bond lengths	/A (esd ~ 0.1 Å)	Bond angles	/° (esd ~ 2°)
Cu-NH_2	2.1	NH_2-Cu-N (His1)	93
Cu-N (His1)	2.0	NH_2-Cu-O (Asn41)	95
Cu-O (Asn41)	2.7	N(His1)-Cu-N(Asn41)	153

Supplementary Table 8. Global pLDDT values for *Mm*Ang-2a models.

Model	Model 0 pLDDT	Model 1 pLDDT	Model 2 pLDDT	Model 3 pLDDT	Model 4 pLDDT
Apo *Mm*Ang-2a-HupE/UreJ	78.8	79.1	80	78.5	80.2
2xCu *MmAng-2a*-HupE/UreJ*	75.62	76.09	75.99	75.45	75.90

* There are 2 Cu atoms bound

Supplementary Table 9. List of bond distances at the copper in the Cu-loaded model of *Mm*Ang-2a.

Metal- Nitrogen	Model 0 bond distance	Model 1 bond distance	Model 2 bond distance	Model 3 bond distance	Model 4 bond distance
Cu-NH_2 (His 1)	1.9 Å	0.8 Å	2.4 Å	-	1.2 Å
Cu-N (His 1)	1.1 Å	2.6 Å	1.3 Å	-	3.3 Å
Cu-O (Asp 33)	2.8 Å	1.9 Å	1.5 Å	-	1.8 Å
Cu-N (His 194)	2.1 Å	2.1 Å	2.0	2.1 Å	2.0 Å
Cu-N (His 201)	2.1 Å	2.1 Å	2.5	2.3 Å	2.2 Å
Cu-N (His 229)	2.0 Å	2.0 Å	2.0	1.9 Å	1.9 Å

	Model 0 Scores	Model 1 Scores	Model 2 Scores	Model 3 Scores	Model 4 Scores
ipTM	0.53	0.52	0.51	0.50	0.50
pTM	0.52	0.51	0.50	0.49	0.49
Ranking score	0.59	0.58	0.57	0.56	0.56

Source Data 3 – Uncropped SDS-PAGE Photos

Gel from Supplementary Figure 3 C.

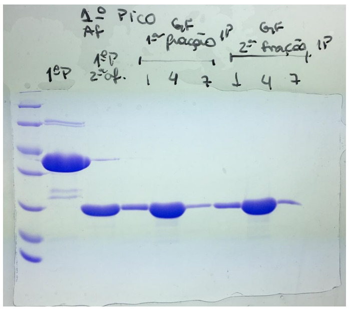

Gel from Supplementary Figure 9 C.

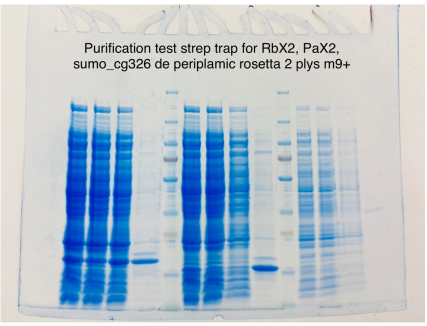

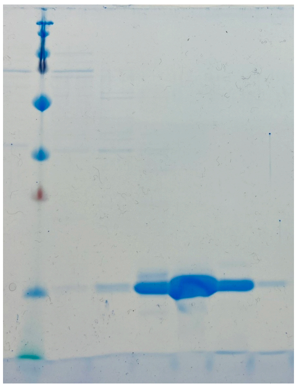

Gel from Supplementary Figure 16 C and 19 C.

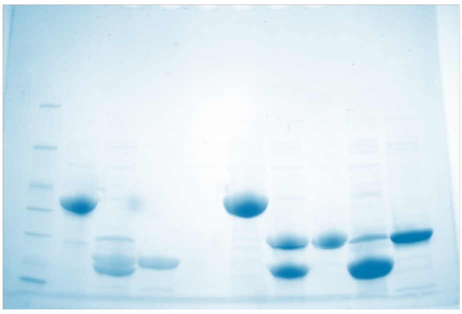

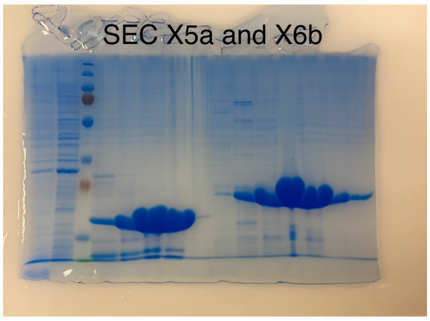

Supplementary References

1. Kittl, R., Kracher, D., Burgstaller, D., Haltrich, D. & Ludwig, R. Production of four Neurospora crassa lytic polysaccharide monooxygenases in Pichia pastoris monitored by a fluorimetric assay. *Biotechnol. Biofuels* **5**, 79 (2012).
2. Breslmayr, E. *et al.* A fast and sensitive activity assay for lytic polysaccharide monooxygenase. *Biotechnol. Biofuels* **11**, 79 (2018).
3. Ipsen, J. Ø., Hallas-Møller, M., Brander, S., Lo Leggio, L. & Johansen, K. S. Lytic polysaccharide monooxygenases and other histidine-brace copper proteins: structure, oxygen activation and biotechnological applications. *Biochem. Soc. Trans.* **49**, 531–540 (2021).
4. Frandsen, K. E. H. *et al.* Identification of the molecular determinants driving the substrate specificity of fungal lytic polysaccharide monooxygenases (LPMOs). *J. Biol. Chem.* **296**, 100086 (2021).
5. Terrasan, C. R. F. *et al.* Deletion of AA9 Lytic Polysaccharide Monooxygenases Impacts A. nidulans Secretome and Growth on Lignocellulose. *Microbiol. Spectr.* **10**, e02125-21 (2022).
6. Rodionov, D. A., Hebbeln, P., Gelfand, M. S. & Eitinger, T. Comparative and Functional Genomic Analysis of Prokaryotic Nickel and Cobalt Uptake Transporters: Evidence for a Novel Group of ATP-Binding Cassette Transporters. *J. Bacteriol.* **188**, 317–327 (2006).
7. Rawlings, N. D. & Barrett, A. J. [13] Evolutionary families of metallopeptidases. in *Methods in Enzymology* vol. 248 183–228 (Academic Press, 1995).

8. Koonin, E. V. & Tatusov, R. L. Computer Analysis of Bacterial Haloacid Dehalogenases Defines a Large Superfamily of Hydrolases with Diverse Specificity: Application of an Iterative Approach to Database Search. *J. Mol. Biol.* **244**, 125–132 (1994).
