# Supplementary material for: Signal-strapping as a protein-sequence search method for the discovery of metalloproteins
Source: Nat Commun. 2025 Oct 20;16:9244. doi: 10.1038/s41467-025-64309-x (PMC12537917; doi:10.1038/s41467-025-64309-x)
Supplement: Supplementary file 2 — Description of Additional Supplementary Files [file 41467_2025_64309_MOESM2_ESM.pdf]

Description of Additional Supplementary Files

File Name: Supplementary Data 1-4
Description: Examples of amino acid sequences retrieved from the NCBI or UniProt databases for DUF4198 (Supplementary Data 1), DUF6702 (Supplementary Data 2), Ang-1 (Supplementary Data 3) and Ang-2 (Supplementary Data 4).

File Name: Supplementary Movie 1
Description: Interdomain dynamic movements of RbAng-1aHupE/UreJ-2. The conformations were assessed from the normal mode analyses of DynaMut web server. The movie file shows the compilation of several vector diagrams, showing the high relative dynamics of the anglerase domain RbAng-1a to that of the HupE/UreJ-2 domain.
